# Parameter identification and sensitivity analysis of a lower-limb musculoskeletal model

**DOI:** 10.3389/fbioe.2025.1566381

**Published:** 2025-04-14

**Authors:** Jinghang Li, Keyi Wang, Yi Yuan, Zhipeng Deng, Yi Lui

**Affiliations:** ^1^ College of Mechanical and Electrical Engineering, Harbin Engineering University, Harbin, Heilongjiang, China; ^2^ Joint Department, Ningbo No.2 Hospital, Ningbo, Zhejiang, China; ^3^ Department of Information and Communication Engineering, School of Engineering, Institute of Science Tokyo, Tokyo, Japan

**Keywords:** Hill-type muscle model, parameter identification, SEMG signal, sensitivity analysis, joint torque estimation

## Abstract

The estimation of joint torque based on wearable sensors is an important content in human–robot interaction research. Despite existing joint torque estimation models providing high accuracy, their application in robotic control is limited due to the number of sensors and real-time output requirements. To address this issue, this paper establishes a knee joint torque estimation model driven by four electromyography (EMG) sensors and proposes a novel method for simplifying musculoskeletal models based on sensitivity analysis. To achieve this, this paper combines multiple advanced Hill-type muscle model components to establish a knee-joint musculoskeletal model that includes four major muscles and employs the genetic algorithm (GA) to identify the model parameters. Then, Sobol’s global sensitivity analysis theory is used to analyze the influence of parameter variations on model outputs, and a sensitivity-based model simplification method is proposed. In addition, a lower-limb physical and biological signal collection experiment without ground reaction force is designed for parameter identification and sensitivity analysis. Finally, based on experimental data from several test subjects, the parameters of each individual’s musculoskeletal model are identified and evaluated, and the sensitivity index of each parameter is calculated to determine the influence of the number of model parameters on the identification performance. The results indicate that the proposed musculoskeletal model can provide individuals with comparable normalized root mean square error (NRMSE) through parameter identification, and the sensitivity-based model simplification method is effective.

## 1 Introduction

In robotic systems involving human–machine interaction, the evaluation or prediction of joint motion or torque is an active area of research (Sitole and Sup, 2023), which is particularly important for rehabilitation, nursing robots, and medical diagnostic devices. Currently, such studies are mainly used to improve the control effects and efficiency of robots (Erden and Tomiyama, 2010). Therefore, balancing accuracy and real-time performance is key to enhancing the actual experience of human–robot systems.

The perception or prediction of human joint torque requires the collection of biological and physical signals during motion, followed by establishing a mapping relationship between sensor signals and joint torque (Hou et al., 2016). With the development of sensor technology, the acquisition and processing technologies for human biological and physical signals have become increasingly mature. Surface electromyography (sEMG) and motion capture system (MoCap) are commonly used to obtain these signals (Bütepage et al., 2018; Downey et al., 2017). sEMG and MoCap are convenient to wear and are non-invasive sensors, which can greatly avoid the impact caused by discomfort. Their fast signal acquisition and transmission make them not only suitable for research on human motion prediction but also can be directly applicable to robot control systems (Ao et al., 2017).

Joint torque prediction based on biological and physical signals is typically achieved through two methods: model-based method and model-free method. The model-based method uses dynamic and biomechanical models to describe the transformation relationship between EMG and joint torque from a mechanistic perspective, while the model-free method treats biological and physical signals as independent or control variables, with joint torque considered an output variable, and uses machine learning techniques to train models (Sitole and Sup, 2023). Both methods have their advantages and disadvantages, and the appropriate method should be selected according to the research objective. Model-free methods use standardized mapping functions to construct black-box models, which require relatively large datasets for sufficient training of the model. In addition, it is necessary to ensure that individuals in the data samples are widely representative to guarantee the generalization ability of the model (Loi et al., 2023). If insufficient data or a lack of individual diversity exists, this may limit the applicability of the trained model, making it difficult to effectively generalize to unseen individuals (Sartori et al., 2016).

Model-based methods usually identify parameters of musculoskeletal or neuromusculoskeletal models and then calculate muscle forces/torques from the activation level obtained from EMG signals. Among these models, Hill’s models or improved Hill’s models are the most versatile and authoritative. For example, Falisse et al. estimated Hill’s model parameters of the individual’s muscle that drives the knee joint by optimizing the formulation of the control problem and determined functional movement sets capable of identifying these parameters (Falisse et al., 2017). Zhao et al. combined Hill’s model and limb dynamics to identify musculoskeletal models driven by EMGs and then estimated wrist joint torques to compute continuous joint angles (Zhao et al., 2020). W. Wang et al. identified sEMG-torque models using particle swarm optimization and coupled gradient methods and corrected errors in real-time using adaptive learning methods (Wang W. et al., 2021). Zhang et al. used the NMS model and LSTM network to estimate joint torques during daily activities based on EMG and kinematic data and trained the model using the reference values obtained via inverse dynamics (Zhang et al., 2023). Although the abovementioned studies were based on Hill’s model, Hill’s model consists of multiple units, each of which has different mathematical descriptions. In order to reduce the dimensions of the model, some studies use the units whose parameters have been fully or partially identified to form Hill’s model, such as using elderly muscle contraction unit parameters for muscle models of individuals of different ages, resulting in loss of interpretability of the identified model. Research that integrates musculoskeletal models with finite element analysis, such as the finite element musculoskeletal (FEMS) framework established by S. Wang et al., also exists, providing a more realistic modeling approach (Wang et al., 2022). They proposed driving the FEMS solely through IMU data, achieving reliable mechanics and secondary kinematics of calculation on the knee joint with low computational costs (Wang S. et al., 2021). Beyond model identification methods, it is also possible to establish a loss function model with the goal of optimizing muscle endurance and then allocate joint torques to each relevant muscle. J. Wen et al. improved the accuracy of this method by conducting secondary optimization on the discrepancies between muscle forces derived from the loss function and those estimated by the musculoskeletal model (Wen et al., 2018). The loss function method typically relies on muscle selection and data quality. To address this issue, Hassaan et al. introduced simplified cost functions based on the Hill model (Ahmed et al., 2024). However, the loss function model needs to solve the optimization problem every time muscle strength is calculated, which is not conducive to applications demanding real-time performance.

When the parameters of a musculoskeletal model are increased, its prediction effect on joint torque is better. However, this will consume excessive computational power to identify the model and may lead to overfitting (Buchanan et al., 2004). In practical applications, musculoskeletal models are often used in robot control, making it unreasonable to identify a large number of parameters online or in real time. Therefore, simplification of the model based on application requirements is necessary to improve its real-time performance. One way to simplify the model is by establishing relationships between parameters and certain information. For example, Saul et al. developed an upper limb model that represents the relationship between joint angles and both normalized muscle fiber lengths and muscle moment arms in the upper limb (Saul et al., 2015). Additionally, there are studies that have employed polynomial functions to model the relationship between tendon lengths and joint angles (Ramsay et al., 2009). Another method is to identify certain parameters in advance which could reduce the number of model parameters. J. Han et al. set the muscle force–velocity relationship in Hill’s model as a constant, establishing a three-parameter muscle model (Han et al., 2015). Pau et al. simplified the force–velocity unit into a polynomial determined solely by muscle contraction speed (Pau et al., 2012). However, the applicability of these methods may not be uniform for different individuals, and balancing the degree of simplification with the universality of a model constitutes a significant challenge. Currently, there are few studies analyzing the parameter sensitivity of musculoskeletal models, i.e., quantifying the impact of parameter perturbations on model outputs. Carol Y. et al. studied and evaluated the sensitivity of 14 parameter changes in Hill’s model in forward dynamics simulation during running and walking (Scovil and Ronsky, 2006). Zhao et al. (2020) also briefly analyzed the parameter sensitivity of wrist joint angle prediction models. However, these studies calculated the parameter sensitivity without simplifying the model based on the sensitivity analysis results.

**FIGURE 1:**
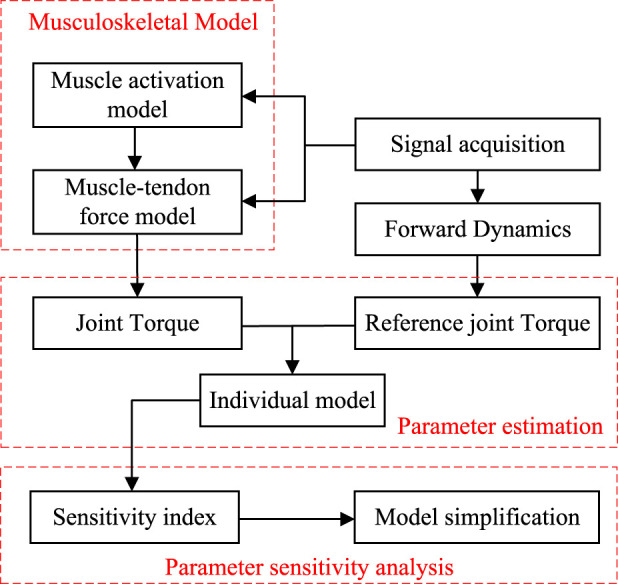
Overall flowchart of this study.

In this paper, an EMG-driven musculoskeletal model and its parameter identification are studied, and a novel model simplification method based on sensitivity is proposed. Section 2 establishes a musculoskeletal model consisting of four major muscles, which combines classical and novel mathematical models in Hill’s model and employs the genetic algorithm (GA) to identify the parameters of the proposed model. Section 3 introduces Sobol’s global sensitivity analysis method to examine the influence of model parameters and their interaction effects on the musculoskeletal model output, with the objective of model simplification. Section 4 explains the physical and biological signal collection experimental process for parameter identification. Section 5 identifies the parameters and sensitivity index of each individual musculoskeletal model and summarizes the impact of model parameter number on the identification process. The overall flowchart of this study is shown in Figure 1.

## 2 Modeling and parameter identification

The single degree-of-freedom knee joint is the research object of this paper. The relationship between sEMG and active knee-joint torque is established through a proposed musculoskeletal model that integrates the muscle activation model with muscle–tendon models. Knee flexion and extension torques are mainly generated by the hamstring and quadriceps muscle groups. However, a musculoskeletal model involving all muscles within these groups would be overly complex, while simultaneously collecting EMG signals from each muscle is neither cost-effective nor conducive to the experiment. Therefore, the following simplifications are made for the proposed musculoskeletal model:

(1) The activation levels of the hamstrings and quadriceps are reflected by four major thigh muscles.
(2) For the hamstring group, the knee flexion torque is approximated as being produced by the long head of the biceps femoris (BF). Since BF has a large muscle volume and is close to the skin surface, it results in a generally high-quality EMG signal. In contrast, the other muscles in the hamstring group are either small or located deep under the skin.
(3) For the quadriceps group, the vastus intermedius (VI) is excluded from the quadriceps, and the knee extension moment is supported by the rectus femoris (RF), vastus lateralis (VL), and vastus medialis (VM). Since VI is located deep beneath the RF and is entirely covered by RF, acquiring its EMG signal is challenging, and its activity is highly synergistic with RF.

Subsequently, the joint torques derived from the dynamics of the lower limb are considered the reference output for the proposed musculoskeletal model, while the collected EMG signals serve as the input to the model. Then, an optimization method is employed to calibrate the individual model parameters.

### 2.1 Musculoskeletal modeling

**FIGURE 2:**
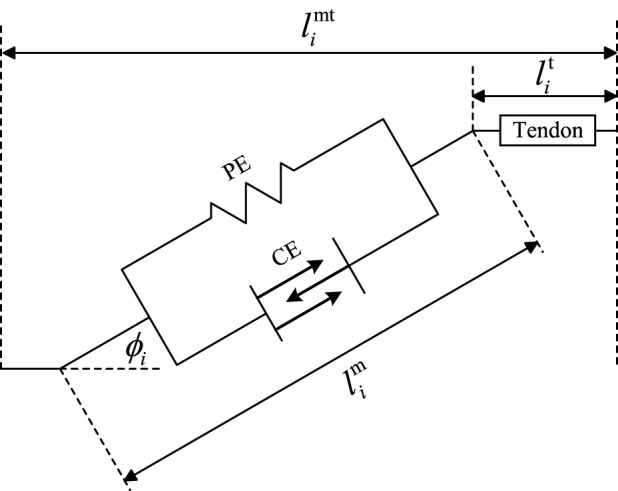
Hill’s muscle model.

Hill’s model is a commonly used and widely accepted mathematical model for calculating muscle–tendon force $F_{i}^{mt}$ in neuromuscular research. It simplifies the muscle fiber as a series–parallel system of the elastic component (PE) and contraction component (CE), and the basic configuration is shown in Figure 2.

In Figure 2, $l_{i}^{mt}$ , $l_{i}^{m}$, and $l_{i}^{t}$ are the muscle–tendon length, muscle fiber length, and tendon length, respectively. The pennation angle $\phi_{i}$ is the angle between the muscle fiber and the tendon, and the feathering angle of any muscle can be expressed as shown in Equation 1:

$$\phi_{i}=\sin^{-1}\left(\frac{l_{o,i}^{\text{m}}\sin\phi_{o,i}}{l_{i}^{\text{m}}}\right),$$ (1)

where $l_{\text{o},i}^{\text{m}}$ and $\phi_{\text{o},i}$ are the optimal fiber length and optimal pennation angle, respectively. It is assumed that tendons on both sides of the muscle move only along a single direction, and the change in tendon length is ignored. The muscle fiber length at any time can be expressed as shown in Equation 2:

$$l_{i}^{\text{m}}=\sqrt{{\left(l_{i}^{\text{mt}}-l_{i}^{\text{t}}\right)}^{2}+{\left(l_{\text{o},i}^{\text{m}}\sin\phi_{\text{o},i}\right)}^{2}},$$ (2)

where $l^{\text{t}}$ is the fixed tendon length.

Tendon force can be expressed as the sum of the active muscle force $F_{\text{CE}}$ and passive force $F_{\text{PE}}$, as shown in Equation 3:

$$F^{\text{mt}}=\left(F_{\text{CE}}+F_{\text{PE}}\right)\cos\phi$$ (3)

The active muscle force $F_{\text{CE}}$ is generated by muscle contraction (CE) and related to the muscle activation, as shown in Equation 4:

$$F_{\text{CE},i}=F_{\text{o},i}^{\text{m}}\left(\;f_{\text{L}}\left({\bar{l}}_{i}^{m}\right)f_{\text{V}}\left({\bar{v}}_{i}^{m}\right)a_{i}\left(t\right)\;\right),$$ (4)

where $F_{\text{o},i}^{\text{m}}$ is the maximum voluntary muscle force; $f_{\text{L}}\left({\bar{l}}_{i}^{m}\right)$ and $f_{\text{V}}\left({\bar{v}}_{i}^{m}\right)$ are the normalized active fiber force length and normalized force–velocity relationships, respectively; and $a_{i}\left(t\right)$ is the muscle activation at time *t*. ${\bar{l}}_{i}^{m}$ and ${\bar{v}}_{i}^{m}$ are normalized fiber length and velocity of muscles, respectively, and calculated using Equation 5:

$${\bar{l}}_{i}^{m}=\frac{l_{i}^{m}}{l_{\text{o},i}^{m}};{\bar{v}}_{i}^{m}=\frac{v_{i}^{m}}{v_{\text{o},i}^{m}},$$ (5)

where $l_{\text{o},i}^{m}$ is the optimal muscle fiber length and $v_{i}^{m}$ and $v_{\text{o},i}^{m}$ are the muscle contraction velocity and optimal muscle contraction velocity, respectively.

The raw EMG signal is normalized and then converted to muscle activation by the following formula given in Equation 6 (Günther et al., 2007):

$$a_{i}\left(t\right)=\frac{e^{Au_{i}\left(t\right)}-1}{e^{A}-1},$$ (6)

where $u_{i}\left(t\right)$ is the normalized EMG signal at time *t* and *A* is a nonlinear coefficient that represents the degree of nonlinearity in this equation and ranges from highly nonlinear (−3) to linear (0.01).

The force–length relationship equation of muscle fibers $f_{\text{L}}\left({\bar{l}}_{i}^{m}\right)$ represents the relationship between length and muscle force during active contraction. It can be expressed as follows (Rockenfeller and Günther, 2017):

$$f_{\text{L}}\left({\bar{l}}_{i}^{m}\right)=\left\{\begin{array}{c} \exp\left(-{\left|\frac{{\bar{l}}_{i}^{m}-1}{\Delta W_{asc}}\right|}^{v_{asc}}\right),\;{\bar{l}}_{i}^{m}\leq 1\;\\ \exp\left(-{\left|\frac{{\bar{l}}_{i}^{m}-1}{\Delta W_{dec}}\right|}^{v_{dec}}\right),\;{\bar{l}}_{i}^{m}>1\; \end{array}\right.,$$ (7)

where $\Delta W_{\text{asc}}$ and $v_{\text{asc}}$ determine the function curve shape when muscle length is less than $l_{\text{o},i}^{m}$ , while $\Delta W_{\text{des}}$ and $v_{\text{dec}}$ determine the function curve shape when muscle length is greater than $l_{\text{o},i}^{m}$ . Equation 7 constructs $f_{\text{L}}\left({\bar{l}}_{i}^{m}\right)$ using an ascending (asc) and a descending (des) standard bell curve. ΔW controls the width of the curve with a range from 0 to 1, and *v* represents its exponent power, generally taken between 2 and 4.

The relationship between muscle force and contraction velocity is shown in Equation 8 (Schutte, 1993):

$$f_{\text{V}}\left({\bar{v}}_{i}^{m}\right)=\left\{\begin{array}{c} {\bar{v}}_{i}^{m}=\frac{v_{i}^{m}}{q^{v}l_{o,i}^{m}}\;\\ \frac{0.3\left({\bar{v}}_{i}^{m}+1\right)}{-{\bar{v}}_{i}^{m}+0.3},\;{\bar{v}}_{i}^{m}\leq 0\;\\ \frac{2.34{\bar{v}}_{i}^{m}+0.039}{1.3{\bar{v}}_{i}^{m}+0.039},\;{\bar{v}}_{i}^{m}>0\; \end{array}\right.,$$ (8)

where $q^{v}$ represents the relationship between $v_{\text{o},i}^{m}$ and $l_{\text{o},i}^{m}$ .

The passive force $F_{\text{PE}}$ generated by PE can be represented as shown in Equation 9 (Thelen, 2003):

$$F_{PE,i}=\left\{\begin{array}{c} 0,\;{\bar{l}}_{i}^{m}\leq 1\;\\ \frac{\exp\left(k_{PE}\left({\bar{l}}_{i}^{m}-1\right)/\varepsilon_{0}\right)-1}{\exp\left(k_{PE}\right)-1},\;{\bar{l}}_{i}^{m}>1\; \end{array}\right.,$$ (9)

where $k_{\text{PE}}$ is a curve-shaped parameter, which is selected as a fixed value according to human age. Generally, young people take $k_{\text{PE}}=5$ and older people take $k_{\text{PE}}=6$ . $\varepsilon_{0}$ represents the maximum muscle tension strain of PE, with a general range of 0.2–0.4.

Substituting Equations 4–9 into Equation 3, a musculoskeletal model $F_{\text{CE},i}\left(l_{i}^{m},v_{i}^{m},u_{i},\lambda \right)$ is proposed, in which $\left[l_{i}^{m},v_{i}^{m},u_{i}\right]$ represents the input to the model, λ is the unidentified parameter vector that can be divided into muscle–tendon parameters and function parameters. The muscle–tendon parameters include optimal fiber length $l_{\text{o},i}^{\text{m}}$ , optimal pennation angle $\phi_{\text{o},i}$ , maximum voluntary muscle force $F_{\text{o},i}^{\text{m}}$ , optimal muscle contraction velocity parameter $q_{i}^{v}$, and tendon length $l_{i}^{\text{t}}$ , while the function parameters include *A*, $\Delta W_{\text{asc}}$ , $v_{\text{asc}}$ , $\Delta W_{\text{des}}$ , $v_{\text{dec}}$, and $\varepsilon_{0}$ . To simplify the number of unidentified parameters, it is assumed that each muscle possesses distinct muscle–tendon parameters, whereas the functional parameters are universal across all muscles.

### 2.2 Model parameter identification method

The mathematical model of a single muscle gives the mapping relationship between muscle activation and single muscle force. Then, the torque generated by a single muscle relative to the knee joint can be expressed as shown in Equation 10:

$$M_{i}=F_{i}^{\text{mt}}r_{i},$$ (10)

where $r_{i}$ is the muscle force arm relative to the knee joint. Furthermore, the total torque of all muscles relative to the knee joint is shown in Equation 11:

$$\tau =\sum M_{\text{f}}-\sum M_{\text{e}},$$ (11)

where $M_{\text{f}}$ represents flexion torque and $M_{\text{e}}$ represents extension torque.

Substituting the established models of four muscles into Equation 11, we obtain a total of 26 unidentified parameters, comprising 20 muscle–tendon parameters and six function parameters. The unidentified parameters are represented in vector form, as shown in Equation 12:

$$\lambda =\left[A,\Delta W_{\text{asc}},v_{\text{asc}},\Delta W_{\text{des}},v_{\text{dec}},\varepsilon_{0},l_{\text{o},i}^{\text{m}},\phi_{\text{o},i},F_{\text{o},i}^{\text{m}},q_{i}^{v},l_{i}^{\text{t}}\right].$$ (12)

Then, the identification of model parameters can be transformed into an optimization problem, as shown in Equation 13:

$$\lambda_{\text{o}}=\min Fun\left(\lambda \right)\text{s}\text{.t}\text{. }\lambda \in \left[\lambda_{\min},\lambda_{\max}\right],$$ (13)

where $\lambda_{\text{o}}$ is the optimal parameter vector, $\left[\lambda_{\min},\lambda_{\max}\right]$ is the range of λ , and Fun(λ) is represented in Equation 14:

$$Fun\left(\lambda \right)=\sqrt{\frac{1}{N}\overset{N}{\underset{n=1}{\sum }}{\left(\tau_{n}-{\hat{\tau }}_{n}\right)}^{2}},$$ (14)

where $\tau_{n}$ and ${\hat{\tau }}_{n}$ represent the estimated joint torque of the model and the reference joint torque, respectively, and *N* is the number of samples. The lower limbs are considered a two-link mechanism, with its inertia matrix and center of mass position derived from an individual musculoskeletal model established in OpenSim. Through inverse dynamics, knee joint torque can be calculated based on the lower limb motion information at each sampling moment, which serves as the reference joint torque ${\hat{\tau }}_{n}$ .

The proposed model requires the identification of 26 parameters and involves the coupling of multiple nonlinear models. Additionally, it is necessary to account for potential noise contamination in the experimental data (fluctuations in EMG signals) and the multimodal optimization problem. Consequently, the GA is employed in this study due to its robust global search capabilities, which are particularly effective in addressing high-dimensional nonlinear optimization problem. The GA’s independence from gradient-based or continuity assumptions makes it well-suited for handling noisy experimental data. Furthermore, the incorporation of crossover and mutation operators within the GA framework mitigates the risk of converging to local optima. λ is encoded as a chromosome in floating-point form, with each parameter within λ acting as a gene. The muscle–tendon parameter values of the individual musculoskeletal model in OpenSim are taken as initial physiological values for λ , and $F_{\text{o},i}^{\text{m}}$ in λ takes a range within 50% of the initial value, while the remaining parameters take a range within 20% of the initial value. The specific parameter ranges are shown in Table 1.

**TABLE 1:** Range of unidentified parameters.

Parameter	Range	Parameter	Range
*A*	[-3, 0.01]	$l_{o,i}^{\text{m}}$	Initial ±50%
$\Delta W_{\text{asc}}$ , $\Delta W_{\text{des}}$	[0.01, 1]	$\phi_{\text{o},i}$	Initial ±20%
$v_{\text{asc}}$ , $v_{\text{dec}}$	[2, 4]	$F_{\text{o},i}^{\text{m}}$	Initial ±20%
$\varepsilon_{0}$	[0.2, 0.6]	$q_{i}^{v}$	[8, 12]
		$l_{i}^{\text{t}}$	Initial ±20%

The range of muscle–tendon parameters does not represent their physiological ranges because the proposed musculoskeletal model is a simplification of the physiological models. During the parameter identification process, some unmodeled muscle functions are represented through mathematical equivalences. The relationship between the parameter values of the proposed model and their physiological counterparts should be interpreted with caution.

## 3 Sensitivity analysis

Since the parameters of each individual’s muscle model are generally different, in applications where real-time performance is not critical or computational resources are sufficient, it is feasible to identify model parameters for each individual to achieve a high-precision model. However, in scenarios with stringent real-time requirements, such as robot control systems or online identification systems, a simplified model with low accuracy loss is needed. To simplify the model and control the accuracy loss of the output, a sensitivity analysis is conducted to assess the impact of each parameter on the model’s output. This allows for the omission of low-sensitivity parameters in the identification process for each individual model, thereby reducing computational complexity without significantly compromising performance. Therefore, this part evaluates the influence of parameters on model output and interaction effects between parameters by using Sobol’s global sensitivity index.

Sobol’s sensitivity analysis requires model parameters as inputs and produces a scalar output. However, the transfer function of the musculoskeletal model is $T:\left[l_{i},v_{i},u_{i},r_{i}\right]\subseteq R^{m}\to \tau$ . Its inputs are biological and physical signals, and the output is the torque of muscle relative to the joint. Furthermore, the influence of model parameters on model outputs can vary significantly depending on the nature of the biological and physical signals. Therefore, a mathematical model that takes model parameters as inputs and produces scalar outputs needs to be constructed. Referring to Equation 14, when the sequence of biological and physical signals collected experimentally is determined, Equation 15 can be obtained:

$$Y=f\left(\lambda \right)=\sqrt{\frac{1}{N}\overset{N}{\underset{n=1}{\sum }}{\left(\tau_{n}\left(\lambda \right)-{\underline{\tau }}_{n}\left(\lambda_{0}\right)\right)}^{2}},$$ (15)

where ${\underline{\tau }}_{n}{\left.\left(\lambda_{0}\right)\right)}^{2}$ is the joint torque obtained through optimal model parameters, $\tau_{n}\left(\lambda \right)$ is the joint torque obtained through arbitrary model parameters, and *Y* represents the output.

*Y* is defined as the disturbance caused to the output when optimal model parameters are replaced with arbitrary model parameters within a specific sequence of biological and physical signals. When the global sensitivity index of a model parameter with respect to *Y* is low, it indicates that changes in this parameter have a minimal effect on *Y*, so this parameter has little disturbance on the output of T and can be set as a constant without identification. Conversely, this parameter will have a greater impact on the output of T and needs to be identified individually for different subjects.

Sobol’s global sensitivity index is calculated using the Monte Carlo method and methods proposed by Sobol and Saltelli (Saltelli et al., 2010; Jansen, 1999), which are based on approximating results from large samples of inputs and outputs. This method not only evaluates the impact of individual input parameters on the output but also quantifies the influence of interactions between different parameters on the output. Additionally, this method provides accurate sensitivity indices, regardless of whether the relationship between model inputs and outputs is linear or highly nonlinear. However, it is important to note that the computational cost of applying this method to high-dimensional models can be significant. Therefore, it is essential to first validate the effectiveness of the model before employing this method.

If Sobol’s method is used to analyze the sensitivity in Equation 15, the first-order sensitivity index can be expressed as Equation 16:

$$S_{\lambda i}=\frac{{\text{Var}}_{\lambda i}\left({\text{E}}_{\lambda ∼i}\left(Y|\lambda_{i}\right)\right)}{\text{Var}\left(Y\right)},$$ (16)

where $\lambda_{i}$ represents the *i*th model parameter, $\lambda_{∼i}$ represents the sample matrix of all parameters, and $\lambda_{i}$ . ${\text{Var}}_{\lambda i}\left(\cdot \right)$ and ${\text{E}}_{\lambda i}\left(\cdot \right)$ represent the variance and mean of (⋅), given a series of $\lambda_{i}$, respectively. The first-order sensitivity index quantifies the impact of parameter $\lambda_{i}$ on the output *Y* but does not account for the effects of $\lambda_{i}$ changing simultaneously with other parameters on the output *Y* (the interaction effect of parameters). Therefore, the global sensitivity index and second-order sensitivity index are also required. The global sensitivity index $ST_{\lambda i}$ can be expressed as Equation 17:

$$ST_{\lambda i}=1-\frac{{\text{Var}}_{\lambda ∼i}\left({\text{E}}_{\lambda i}\left(Y|\lambda_{∼i}\right)\right)}{\text{Var}\left(Y\right)}.$$ (17)

$ST_{\lambda i}$ includes the main effect of the parameter and all interaction effects, which is the primary reference index for evaluating the impact of $\lambda_{i}$ on the output.

The second-order sensitivity index of $\lambda_{i}$ and $\lambda_{j}$ is shown in Equation 18:

$$S_{\lambda ij}=\frac{{\text{Var}}_{\lambda ij}\left({\text{E}}_{\lambda ∼ij}\left(Y|\lambda_{i},\lambda_{j}\right)\right)}{\text{Var}\left(Y\right)}-S_{\lambda i}-S_{\lambda j},$$ (18)

where $\lambda_{ij}$ represents the *i*th and *j*th model parameters, $\lambda_{∼ij}$ represents the sample matrix of all parameters, and $\lambda_{i}$ and $\lambda_{j}$ . $S_{\lambda ij}$ evaluate the impact of simultaneous changes in $\lambda_{i}$ and $\lambda_{j}$ on the output, respectively.

The sensitivity index of each order in Sobol’s method can be obtained analytically by integration methods. However, for high-dimensional models, this method is very complex, and therefore, Monte Carlo methods are often used to approximate the variance of various terms based on a large amount of sample data. Here, Sobol’s sequence was used to sample model parameters, generating two independent sample matrices $A_{P\times 26}$ and $B_{P\times 26}$ , where each row vector represents a sample point of the model parameter and *P* denotes the number of samples. If the *i*th columns of matrixes *A* and *B* are swapped, then $A_{B\left(i\right)}$ and $B_{A\left(i\right)}$ can be obtained; if the *i*th and *j*th columns are swapped, then $A_{B\left(ij\right)}$ and $B_{A\left(ij\right)}$ can be obtained. By substituting each column from the above matrices into Equation 15, output vectors such as f(A) and f(B) can be obtained.

Then, the first-order sensitivity index can be calculated using Equation 19:

$${Var}_{\lambda i}\left(E_{\lambda ∼i}\left(Y|\lambda_{i}\right)\right)=\frac{1}{P}\overset{P}{\underset{p=1}{\sum }}f{\left(B\right)}_{p}\left(f{\left(A_{B\left(i\right)}\right)}_{p}-f{\left(A\right)}_{p}\right),$$ (19)

where ${\left(\cdot \right)}_{p}$ is the *p*th element of the output vector (⋅) .

The variance term in the global sensitivity index is shown in Equation 20:

$${Var}_{\lambda ∼i}\left(E_{\lambda_{i}}\left(Y|\lambda_{∼i}\right)\right)=\frac{1}{P}\overset{P}{\underset{p=1}{\sum }}f{\left(A\right)}_{p}\left(f{\left(A\right)}_{p}-f{\left(A_{B\left(i\right)}\right)}_{p}\right)$$ (20)

The variance term in the second-order sensitivity index is shown in Equation 21:

$${Var}_{\lambda ij}\left(E_{\lambda ∼ij}\left(Y|\lambda_{i},\lambda_{j}\right)\right)=\frac{1}{P}\overset{P}{\underset{p=1}{\sum }}f{\left(A\right)}_{p}f{\left(B_{A\left(ij\right)}\right)}_{p}-f_{0}^{2},$$ (21)

where $f_{0}$ is the mean of the output vector. The mean and variance of the output are shown in Equation 22:

$$\left\{\begin{array}{c} f_{0}=\frac{1}{P}\overset{P}{\underset{p=1}{\sum }}f{\left(A\right)}_{p}\;\\ Var\left(Y\right)=\frac{1}{P}\overset{P}{\underset{p=1}{\sum }}f{{\left(A\right)}_{p}}^{2}-{f_{0}}^{2}\; \end{array}\right..$$ (22)

Calculations are required between each pair of vectors in the individual model to obtain all second-order sensitivity indices. Since there exist multiple individual models in this study, which will greatly increase the computational load, we first consider the global interaction index, as shown in Equation 23:

$$IN=1-\overset{26}{\underset{i=1}{\sum }}S_{\lambda i}.$$ (23)

When *IN* is large, it indicates that there are significant interaction effects between the model parameters. Then, the parameter interaction index ${IN}_{i}$ can be calculated using Equation 24:

$$IN_{i}=ST_{\lambda i}-S_{\lambda i}.$$ (24)

If ${IN}_{i}$ is above a specific threshold, the calculation of second-order sensitivity index is considered.

Sobol’s method can calculate a higher-order sensitivity index but is limited by the amount of computation. After calculating the parameter sensitivity index for an individual musculoskeletal model, when ranking the importance of parameters, the global sensitivity coefficient ${ST}_{\lambda i}$ should be considered the primary criterion. If ${ST}_{\lambda i}$ is very small, this indicates that the parameter $\lambda_{i}$ not only has a minimal impact on the output but also exhibits little interaction effects with other parameters. Then, $\lambda_{i}$ can be treated as a fixed value for model simplification. If $S_{\lambda ij}$ is large, it indicates that different combinations of parameters $\lambda_{i}$ and $\lambda_{j}$ will have a greater impact on the output, and both parameters need to be identified simultaneously to ensure the accuracy of the model.

After parameters for multiple subjects’ models are systematically identified, sensitivity analysis can be performed on each individual’s musculoskeletal model. The basic idea of the sensitivity-based model simplification method is to start with the simplest model, in which the unidentified parameters should include all high-sensitivity parameters and those with significant interaction effects. By gradually increasing the number of unidentified parameters in the simplest model according to the descending order of the sensitivity index, the changes in model performance during this process can be examined to obtain an appropriate simplified model. For the musculoskeletal model proposed in this paper, the specific steps of the simplification method are as follows:

(1) For the first-order sensitivity index, calculate the mean of the sensitivity index for each parameter across different individual models. Set a threshold, and consider parameters with a mean index higher than this threshold as high-sensitivity parameters.
(2) For individual models with interaction effects, set a threshold, and consider parameters with a second-order sensitivity index higher than this threshold as high-interaction effect parameters.
(3) Consider the high-sensitivity and high-interaction effect parameters as unidentified parameters while treating the remaining parameters as constants, thereby forming the simplest model.
(4) Employ the GA to identify the parameter of the simplest model, evaluate, and record its performance. Subsequently, based on the mean first-order sensitivity index, gradually increase the number of unidentified parameters in the simplest model, identify the parameters, and record the model’s performance accordingly.
(5) Based on the performance required for musculoskeletal model applications, the final simplified model can be determined.

These steps are primarily targeted at the model proposed in this paper. When other models employ the sensitivity-based simplification method, adjustments need to be made according to the aforementioned basic idea. Additionally, the GA operators used in the method differ from those utilized in Section 2.2. Considering that the number of unidentified parameters in this process might be too small, employing floating-point chromosomes for crossover and mutation might lead to convergence at a local optimum. Therefore, simulated binary crossover (SBX) and polynomial mutation operators are used to enhance the randomness during the iteration process of the GA.

## 4 Experiment

**FIGURE 3:**
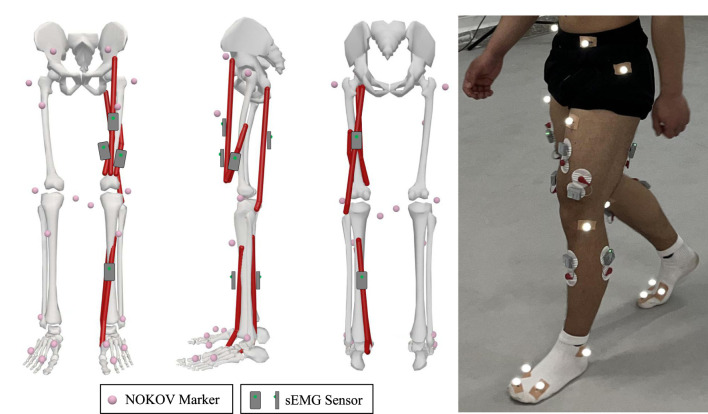
Arrangement of sensors: 20 markers are attached on the subject’s lower limb, four EMG sensors are placed on major muscles of the knee joint for this study, and two EMG sensors are used for other studies.

The raw EMG signals are collected using the wireless surface electromyography analysis system (YW-Wireless) from Beijing Zhitong Huayu Technology Co., Ltd. The wireless sEMG sensors are placed on the skin closest to the target sampling muscles, with a sampling frequency of 2,000 Hz and a receiver bandwidth set at 7 Hz–1,000 Hz. The lower limb motion information is collected using the NOKOV motion capture system, which has a sampling frequency of 200 Hz and consists of 12 cameras and 20 markers. All sensor positions in the lower limbs are shown in Figure 3.

The subjects were asked to perform static standing, maximum voluntary contraction (MVC) tests, and hip joint internal/external and flexion/extension movements. Static standing movement is used for constructing individual musculoskeletal simulation models in OpenSim. The MVC tests are conducted according to the experiment protocol given by Moreira et al. (2021) and include tests for quadriceps and hamstrings muscles, which are completed in sitting and lying positions, respectively. The MVCs are used for calculating standardized EMG signals. Hip joint internal/external and anterior/posterior extension movements require the subject to first stand on one leg so that the tested leg is suspended; then, the subject tries to keep the thigh and calf in a straight line with the knee angle unchanged, followed by performing hip joint adduction, abduction, flexion, and extension, and finally returning to a standing position. This action can ensure single-leg suspension, thus simplifying the need for ground reaction force sensors, while ensuring the contraction and extension of thigh muscles and allowing the knee joint to bear a certain torque. When the participants performed the actions, the raw signal from two kinds of sensors was synchronously collected using XINGYING software of the NOKOV motion capture system, and after interpolation of distorted points, the position data of marker points in a ground coordinate system and sEMG signal data were exported.

**FIGURE 4:**
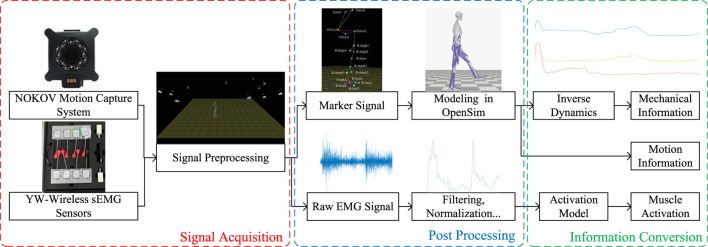
Schematic diagram of signal processing.

Considering the variability in the EMG intensity, skeleton, and muscle geometry among different individuals, it is necessary to perform normalization and calibration based on biological and physical signals. For the EMG signal, the band-pass filter is used to remove noise. A second-order Butterworth band-pass filter with cutoff frequencies at 20 Hz and 450 Hz is used, and a fourth-order Butterworth low-pass filter with a corner frequency at 6 Hz is then applied. Following this, the absolute value of the filtered EMG signal is taken and then divided by the maximum EMG amplitude recorded during MVC, thereby obtaining the normalized EMG signal. For the calibration of skeleton and muscle geometry, along with using medical imaging (such as MRI), which offers high precision but is complex and costly, one approach is to create personalized musculoskeletal models and motion simulations in OpenSim. This method only requires motion capture data to obtain geometric data. By using motion capture data from a standing position to scale the simulation model in OpenSim, one can obtain approximate skeletal geometry data for the individual. Then, based on motion capture data during movement, action simulations are performed to derive specific time-varying values of tendon lengths and muscle moment arms, serving as muscle geometry data. After calibrating the musculoskeletal model, it is then possible to analyze the reference joint torque through the lower limb dynamics. The schematic diagram illustrating the biological and physical signal processing methods is shown in Figure 4.

## 5 Simulation and result analysis

### 5.1 Verification of the proposed model

**FIGURE 5:**
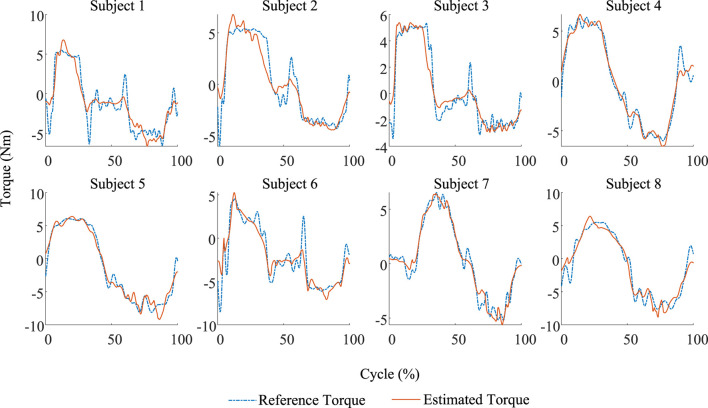
Comparison between the estimated results and the reference of eight subjects in the validation trial. Each identified model exhibits good accuracy.

In this study, eight subjects were selected to collect the biological and physical information on lower limb movement, and the parameters of each individual’s musculoskeletal model are identified. The identified model is vilified by the validation set composed of signals collected from a single cycle of lower limb movements using NRMSE and $R^{2}$ . The NRMSE evaluates the amplitude difference between the model output and the reference results, while $R^{2}$ assesses the correlation between the model output and the reference results. The smaller the NRMSE value and the greater the $R^{2}$ value, the better the model performance. Figure 5 shows the comparison of knee joint torque curves calculated by the identified models with the reference joint torque curve in the validation set. To eliminate differences in movement speed between individuals in Figure 5, the movement cycle (from the start to the end of the movement) is standardized into a normalized timeline ranging from 0% to 100%, denoted as Cycle (%). Table 2 presents the evaluation parameters of the result.

**TABLE 2:** NRMSE and $R^{2}$ in the validation trial. The model performances of S1 and S6 are the poorest, whereas those of S4 and S7 are the best.

Subject	NRMSE (%)	$R^{2}$	Subject	NRMSE (%)	$R^{2}$
S1	11.226	0.8590	S5	6.2660	0.9721
S2	10.702	0.8917	S6	10.482	0.8357
S3	10.320	0.9052	S7	5.4358	0.9656
S4	5.9170	0.9709	S8	8.6556	0.9409

**FIGURE 6:**
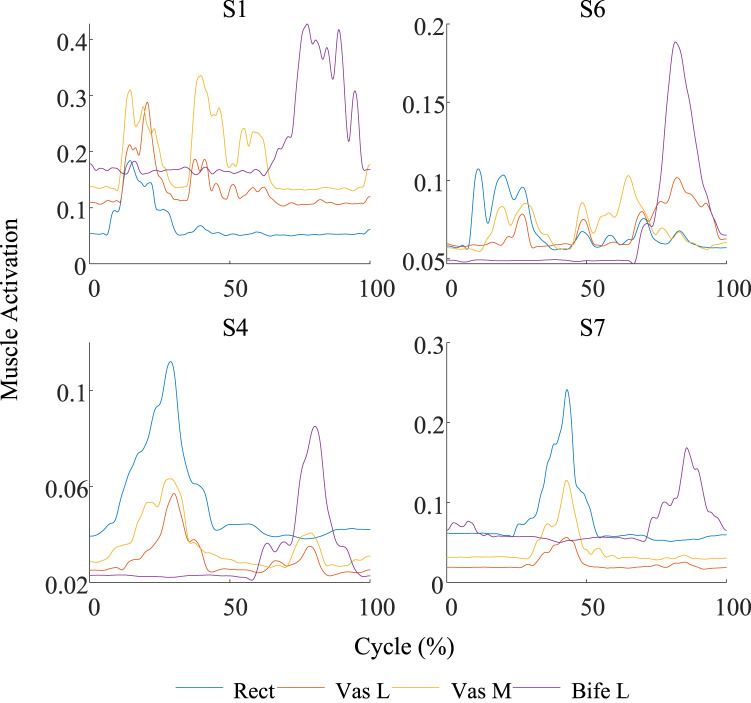
Comparison of the muscle activation curves between the worst group (S1 and S6) and the best group (S4 and S7). The curve fluctuation of the worst group is significantly greater than that of the best group.

According to the evaluation results, the NRMSE and $R^{2}$ of Subject 1 (S1) and Subject 6 (S6) are worse than those of other subjects. As shown in Figure 5, the reference torque of S1 and S6 exhibits abrupt changes near 50% of the motion cycle. The transition from positive torque to negative torque indicates that the lower limb underwent distinct end and start phases of movement. In addition, from the perspective of the model input, Figure 6 compares the muscle activation changes between the worst group (S1 and S6) and the best group (S4 and S7). It can be observed that the fluctuations in muscle activations in the worst group are significantly more frequent and less distinct than those in the best group. Therefore, the superposition of these two factors may have contributed to the poor final evaluation results.

Compared with the results evaluated by Zhang et al. (2023) and Wang W. et al. (2021), where the former uses both the identification model and LSTM model and the evaluation results are NRMSEs of 15.2% and 6.0%, the proposed model in this study demonstrates better results than the identification model proposed by Zhang et al. (2023), but is inferior to the LSTM model. The latter also employed an identification method, but with the addition of an error prediction mechanism, it results in a single individual model evaluation RMSE of 1.14. If the proposed model in this study used RMSE to evaluate the performance, the best and worst results are 0.6376 and 1.3555, respectively, indicating that the proposed model is superior in some individuals to the model proposed by Wang W. et al. (2021). However, considering that the model proposed by Wang W. et al. (2021) did not validate across multiple individuals, the comparison can only serve as a preliminary reference.

The above results demonstrate that the proposed model can accurately estimate knee joint torque based on biological and physical signals, exhibiting a certain level of accuracy under controllable input signal noise.

### 5.2 Parameter sensitivity analysis result

In this section, global sensitivity analysis is conducted on the musculoskeletal model of each individual, followed by model simplification based on the sensitivity and, finally, an evaluation of the performance of the simplified model. It should be noted that sensitivity analysis can be directly applied to the proposed model without parameter identification. However, conducting sensitivity analysis without first validating the effectiveness of the model would be meaningless. Furthermore, to compare the accuracies of the original and simplified models, sensitivity-based model simplification is performed on each identified model.

According to the specific values of all individual model parameters, the mean of each parameter is shown in Table 3. This value will be used as the fixed parameter value in the simplified model.

**TABLE 3:** Mean of identified parameters.

Function parameter	Value	Function parameter	Value
$A/\lambda_{1}$	−1.3895	$\Delta W_{\text{asc}}/\lambda_{2}$	0.3514
$v_{\text{asc}}/\lambda_{3}$	2.7315	$\Delta W_{\text{des}}/\lambda_{4}$	0.4015
$v_{\text{dec}}/\lambda_{5}$	2.5282	$\varepsilon_{0}/\lambda_{6}$	0.3687

Muscle–tendon parameter	RF	VL	VM	BF
$l_{\text{o},i}^{\text{m}}\left(\text{m}\right)\text{ / }\lambda_{7}∼\lambda_{10}$	0.0957	0.0667	0.1049	0.1020
$\phi_{\text{o},i}\left(\text{rad}\right)\text{ /}\lambda_{11}∼\lambda_{14}$	0.0946	0.0838	0.1028	0.2419
$F_{\text{o},i}^{\text{m}}\left(\text{N}\right)\text{ / }\lambda_{15}∼\lambda_{18}$	1196.8	1779.8	1000.7	1437.7
$q_{i}^{v}\text{ / }\lambda_{19}∼\lambda_{22}$	13.794	8.9608	7.7009	11.862
$l_{i}^{\text{t}}\text{ / }\lambda_{23}∼\lambda_{26}$	0.4278	0.2006	0.1538	0.4466

**FIGURE 7:**
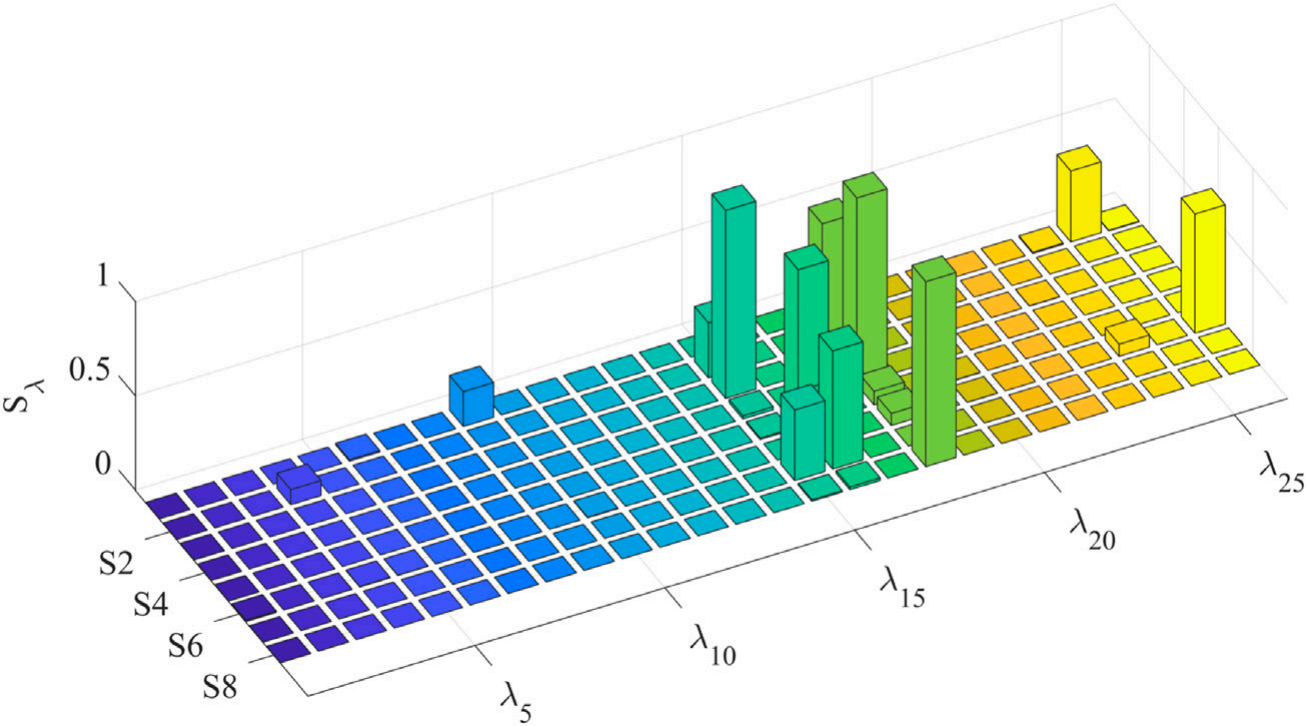
First-order sensitivity index of parameters in each individual model.

**FIGURE 8:**
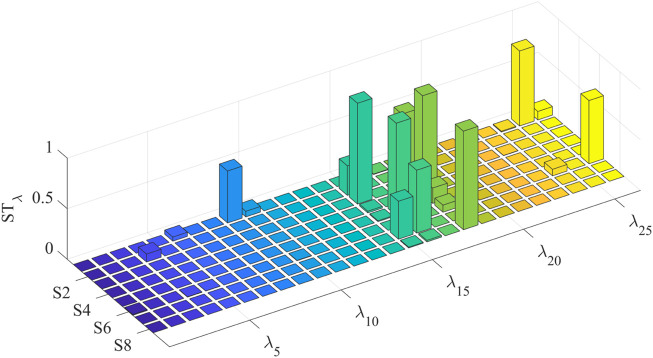
Global sensitivity index of parameters in each individual model.

To simplify the model based on the sensitivity of the parameters to the model output, the first-order and global sensitivities of each individual identification model are calculated separately. The first-order sensitivity index of the eight individual models is shown in Figure 7. For multiple subjects, the identified model shows high first-order sensitivity to maximum voluntary muscle force $F_{\text{o},i}^{\text{m}}$ $\left(S_{\lambda i}\leq 0\text{.}9973\right)$ , followed by fixed tendon length $l_{i}^{\text{t}}$ $\left(S_{\lambda i}\leq 0\text{.}6811\right)$ . The first-order sensitivity index of $\Delta W_{\text{des}}$ is evident only in the model of S2 $\left(S_{\lambda 4}=0\text{.}0802\right)$ , and the first-order sensitivity indices of other parameters are relatively low. The global sensitivity index is shown in Figure 8; compared with global sensitivity and first-order sensitivity, their distributions within the individual model parameters are essentially the same, so it can be inferred that there are weak interaction effects between high-sensitivity parameters and low-sensitivity parameters.

**FIGURE 9:**
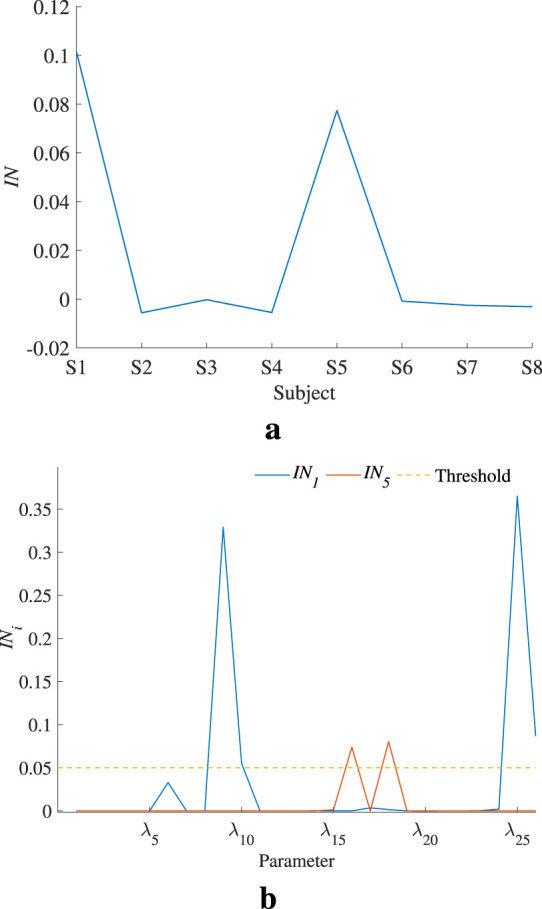
Global and parameter interaction indices. **(a)** indicates that S1 and S5 models have an obvious interaction effect. **(b)** reveals which parameter’s second-order sensitivity index should be calculated. **(a)** Global interaction index of each individual model. **(b)** Parameter interaction index in S1 and S5.

**FIGURE 10:**
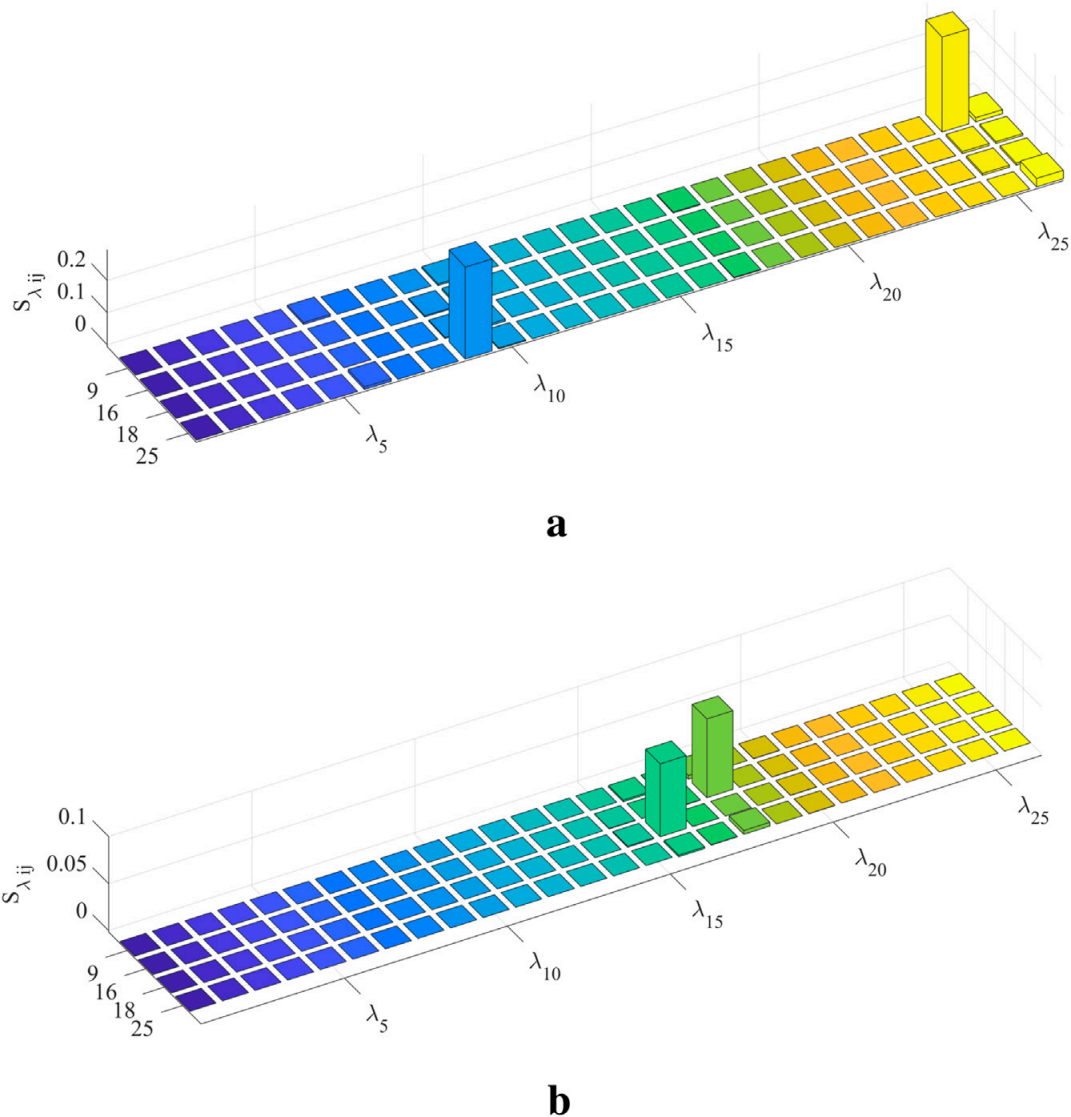
Second-order sensitivity index. **(a)** $S_{\lambda ij}$ for S1. **(b)** $S_{\lambda ij}$ for S5.

To accurately judge the interaction effect between model parameters, the global interaction index *IN* is calculated and shown in Figure 9a. Only the models of S1 and S5 show significant parameter interaction effects. The parameter interaction index ${IN}_{i}$ for the models of S1 and S5 is shown in Figure 9b. If a threshold for ${IN}_{i}$ is set to 0.05, then the second-order sensitivity coefficients of $\lambda_{9}$ , $\lambda_{16}$ , $\lambda_{18}$, and $\lambda_{25}$ should be checked. Figure 10a illustrates the second-order sensitivity index for S1, demonstrating a clear interaction effect between $\lambda_{9}$ and $\lambda_{25}$ . Figure 10b shows the second-order sensitivity index for S5, also revealing a clear interaction effect between $\lambda_{16}$ and $\lambda_{18}$ . This proves that there is no obvious interaction effect between the other parameters, except for the aforementioned four parameters.

**FIGURE 11:**
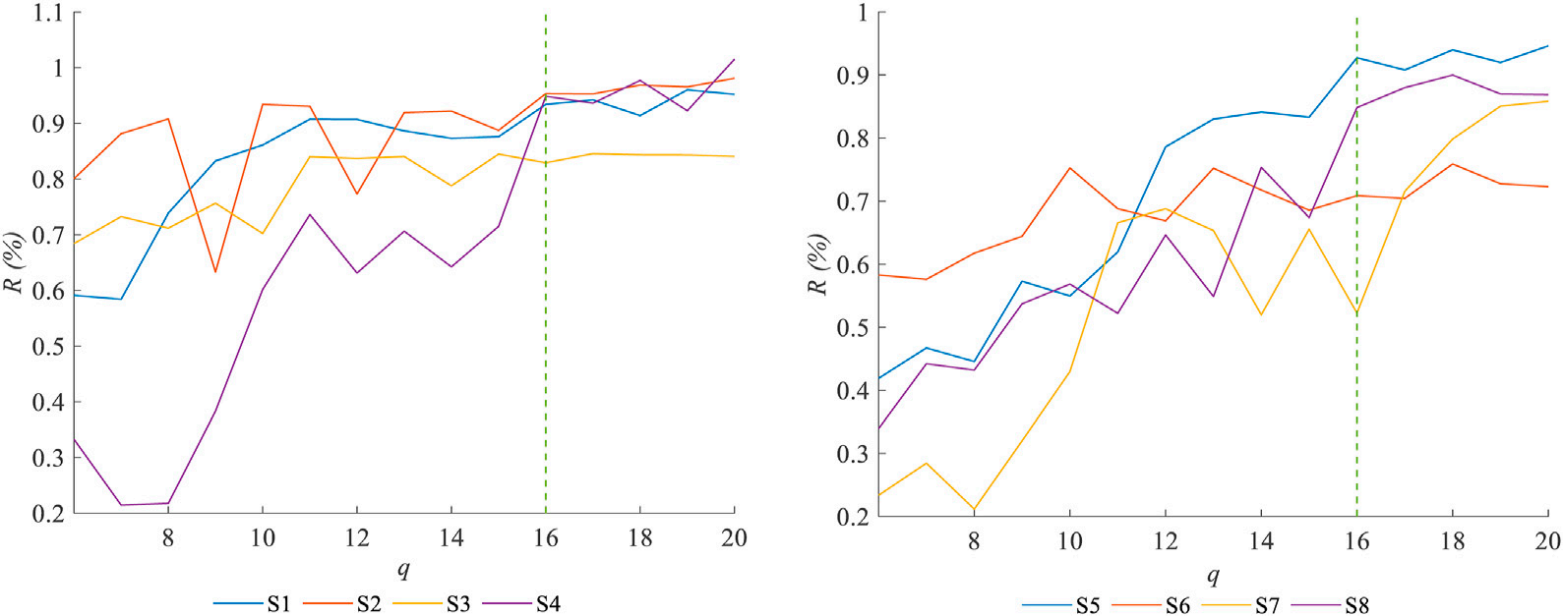
Variation curve of R relative to q .

Based on the results of sensitivity analysis at each order, the proposed model can be simplified. After calculating the mean value of the global sensitivity index for each parameter, the model parameters are arranged in a descending order based on these mean values. The first *q* parameters are selected with the highest sensitivity as identification objects, and then, *q* is incrementally increased to analyze the relationship between model accuracy and the number of identification objects. Additionally, when *q* takes its minimum value, the identification objects should at least include $\lambda_{9}$ , $\lambda_{16}$ , $\lambda_{18}$, and $\lambda_{25}$ , considering the interaction effects among them. Therefore, we take *q* = 4 as the minimum value. The GA is used to identify the parameters of the simplified model, and the ratio of the simplified model’s precision compared to the original model’s precision is expressed as Equation 25:

$$R=\frac{{RMSE}_{O}}{{RMSE}_{S}},$$ (25)

where ${RMSE}_{O}$ is the RMSE of the original model and ${RMSE}_{S}$ is the RMSE of the simplified model. *R* represents the accuracy of the simplified model relative to the original model, indicating that the simplified model can achieve R×100% of the original model’s accuracy. As *q* increases, the change curve of *R* is shown in Figure 11.

It is noted that when q>16 , increasing the number of parameters for most individual models yields minimal improvements in the accuracy. At q=16 , except for Subject 7 and Subject 6, the number of parameters to be identified for the simplified individual models is reduced by approximately 40%, with the accuracy loss represented by the R value being less than 20%. Thus, using the model with q=16 as a simplified model for subsequent research can be considered. In addition, some individual models in Figure 11 show a decrease in *R* as *q* increases. This is because the GA used in the identification process limits the maximum number of iterations to 1,000, which may prevent high-dimensional models from reaching an optimal solution, resulting in a worse identification effect than that of low-dimensional models. Therefore, when applying the simplified model, factors such as computing power, precision, and efficiency should be comprehensively considered. The results of this part provide certain ideas for a more in-depth study on musculoskeletal model simplification.

## 6 Discussion

In this section, the limitations of this study are discussed, along with potential ways to address these limitations and enhance the performance of lower-limb musculoskeletal models.

This study proposes a musculoskeletal model of the knee joint by integrating four major muscles. The selection of these muscles is mainly based on the ease of signal acquisition and related research on EMG signal collection (Moreira et al., 2021; Wei et al., 2023). However, the movement and stability of the knee joint are achieved through the coordinated action of multiple muscles. Although parameter identification compensates for unmodeled muscle functions through mathematical equivalence, the choice of different muscles may influence the musculoskeletal model’s performance. The specific impact and methods for selecting the optimal muscle combination require further investigation. Additionally, there is a structural discrepancy between the proposed model and biological models, which may result in muscle parameters falling outside biological ranges. A study has proposed using measured muscle synergies to estimate unmeasured muscle excitations (Ao et al., 2020). Combining this approach with the proposed musculoskeletal mechanics model could potentially resolve this issue and improve model performance.

The proposed model simplification method is generally applicable to musculoskeletal models. However, it is based on statistics and samples, and its computational cost may be high for high-dimensional models. Moreover, insufficient sample size or suboptimal sampling ranges may lead to misleading conclusions, thus requiring further validation of its applicability in other high-dimensional musculoskeletal models.

The data used in this study were obtained from experiments involving foot suspension movements, which eliminated the need for ground reaction force measurement devices. However, this experimental setup cannot generate data for activities, such as gait or stair climbing. In addition, the EMG signals from public databases do not fully match those of the muscles in the proposed musculoskeletal model. Therefore, the feasibility of the identified model for more types of movements needs to be further verified. In future research, we plan to use robots to apply load forces to human subjects while estimating the force feedback through robot sensors, enabling online/offline identification of lower-limb musculoskeletal models across various movements, with the ultimate goal of applying the identified model to robotic control systems.

## 7 Conclusion

This paper investigates parameter identification and sensitivity analysis of a lower-limb musculoskeletal model, proposing a novel sensitivity-based model simplification method. The constructed musculoskeletal model utilizes only four major muscles, and the identified parameters of multiple individual neuromusculoskeletal models through the GA exhibit good accuracy. By applying Sobol’s global sensitivity analysis to the proposed model, this study systematically reveals the impact of model parameters on the accuracy of joint torque predictions and realizes the objective reduction in the number of unidentified parameters based on the sensitivity. Experimental validation demonstrates that the sensitivity-based simplification method effectively balances the accuracy of torque estimation with the number of unidentified parameters. This study lays a methodological foundation for human–machine interaction control in rehabilitation robotics.

## Data Availability

The raw data supporting the conclusions of this article will be made available by the authors, without undue reservation.
